# Granulicatella adiacens Causing a Parapharyngeal Abscess in a 10-Month-Old Infant: A Rare-Case Report and Literature Review of Deep Neck Infections (DNIs) in Children

**DOI:** 10.7759/cureus.42144

**Published:** 2023-07-19

**Authors:** Saja Tahir, Rand Hasanain, Walid Abuhammour, Ajay P Dsouza, Rubina Lone, Safeena Kherani

**Affiliations:** 1 Pediatrics, Al Jalila Children's Speciality Hospital, Dubai, ARE; 2 Infectious Diseases, Al Jalila Children's Speciality Hospital, Dubai, ARE; 3 Radiology, Al Jalila Children's Speciality Hospital, Dubai, ARE; 4 Laboratory Medicine, Al Jalila Children's Speciality Hospital, Dubai, ARE; 5 Otolaryngology - Head and Neck Surgery, Al Jalila Children's Speciality Hospital, Dubai, ARE

**Keywords:** pediatric infectious disease, pediatric, granulicatella adiacens, deep neck infection, parapharyngeal abscess

## Abstract

*Granulicatella adiacens* is a rare variant of the Streptococcus bacteria. When isolated, *G. adiacens* has been described in cases of endocarditis and bacteremia, but less commonly seen in isolated pyogenic infections. We report a case of a parapharyngeal abscess caused by *G. adiacens* in an otherwise healthy 10-month-old infant, which was successfully treated with antibiotics and surgical drainage. To the best of our knowledge, this is the first described case of a pediatric deep soft tissue neck infection caused by *G. adiacens* with one other report in an adult. Additionally, of all localized infections from this bacteria, this is only the second reported case in the pediatric population. We also include an evidence-based literature review of the clinical presentation, microbiology, imaging modalities, and management approach to deep neck infections (DNIs).

## Introduction

In spite of deep neck infections (DNIs) being relatively uncommon in pediatrics, they account for significant morbidity in children. Early recognition and accurate diagnosis can aid in avoiding life threatening complications such as airway obstruction, sepsis, and mediastinitis [1].

The most common pathogens isolated in DNI are typically polymicrobial and include group A *Streptococcus*, *Staphylococcus aureus*, and oropharyngeal anaerobic bacteria [2]*. Granulicatella adiacens* is a catalase and oxidase negative facultative anaerobic gram positive cocci. Although *G. adiacens* is part of the oral, gastrointestinal, and urogenital normal flora, it is rarely implicated or detected in infections as it requires specific detection and culturing techniques [3]. When reported, it has typically been described as a cause of bacteremia and endocarditis in adults with co-morbidities [4]. More rarely, it has been reported in localized pyogenic infections of the suprapatellar region and elbow joint, hip joint, abdomen, orbit, mandible, spine, and dentition [3,5-10].

Here we report a rare case of a parapharyngeal abscess caused by *G. adiacens* Streptococcus variant in an otherwise healthy child. To our knowledge, this is the first described case of a pediatric deep neck space infection caused by this organism with one other report in an adult [11]. We also present an evidence-based literature review of the clinical presentation, microbiology, imaging modalities, and management approach to DNIs.

## Case presentation

An otherwise healthy 10-month-old girl was admitted in hospital with fever and coryza. Specifically, she had six days of high-grade fever associated with four days of snoring, nasal congestion, drooling, and reduced feeding. She was born at full term after an uncomplicated pregnancy and delivery. Her immunizations were up to date, and she did not have any prior infections, hospital admissions, or developmental issues.

On examination, she looked unwell despite stable vital signs. She had moderate coarse inspiratory stridor with drooling without signs of respiratory distress. She was maintaining her normal saturation on room air with no oxygen support. Palpation of the neck revealed a firm, tender left neck mass at the mandibular angle measuring 2 cm x 4 cm with surrounding shotty lymphadenopathy. Oral cavity examination revealed a left posterior pharyngeal bulging fullness extending to the posterior pillar with a midline shift of the uvula to the right.

Laboratory tests revealed an elevated white blood cell (WBC) count of 26,000/µL (65% neutrophils, 17% lymphocytes). The inflammatory markers were significantly raised with C-reactive protein (CRP) level of 366 mg/L and an erythrocyte sedimentation rate of 108 mm/hr. Kidney and liver function blood tests were normal.

**Figure 1:**
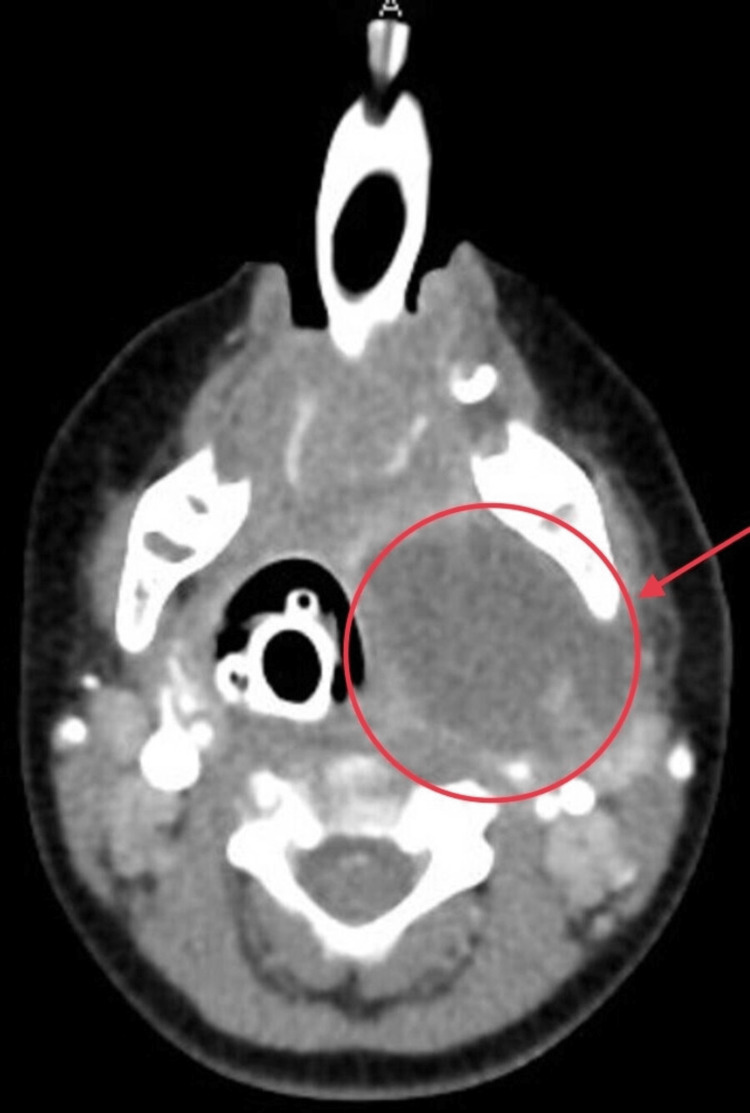
Axial CT scan of the neck with contrast. Red arrow points at a rim-enhancing 2.5 cm x 3.8 cm x 4.0 cm left parapharyngeal abscess with deviation of the airway.

Neck radiograph (X-ray) showed increased pre-vertebral soft tissue swelling. Screening ultrasound revealed a 3.8 cm x 3.4 cm x 3.7 cm left angle of mandible lesion with heterogeneous sluggish content without colour uptake on colour flow examination. There was displacement of the great vessels posteriorly. The overall impression was of liquefactive and phlegmonous lymphadenitis. CT scan of the neck with contrast confirmed a rim-enhancing 2.5 cm x 3.8 cm x 4.0 cm left parapharyngeal abscess extending from skull base to the hyoid bone with mild compression and deviation of the airway (Figure 1).

Considering the severity of her infection, empiric IV vancomycin at the dose of 60 mg/kg/day and IV amoxicillin clavulanate at the dose of 90 mg/kg/day in addition to one dose of IV dexamethasone 0.6 mg/kg were administered. In view of the significant size of the abscess and the airway compression, she underwent urgent surgical drainage. Using an intra-oral approach, at least 20 mL of copious purulent fluid was drained.

In terms of microbiology, the culture from the drained purulent fluid revealed heavy growth of *G. adiacens*. Specifically, heavy alpha-hemolytic growth was noted after 48 hours on blood agar with negative catalase and negative oxidase tests. Further testing using a Gram-Positive Identification Card (Vitek2®, BioMérieux) identified *G. adiacens* with 99% probability. The sample was also sent to another laboratory that confirmed *G. adiacens* using Matrix-Assisted Laser Desorption/Ionization Time-of-Flight Mass Spectrometry (MALDI-TOF MS).

The bacteria was susceptible to vancomycin, ceftriaxone, and clindamycin, and was resistant to penicillin, ampicillin and erythromycin. As a result, IV amoxicillin clavulanate and IV vancomycin were discontinued and the patient was started on IV clindamycin at the dose of 40 mg/kg/day. She completed a 10-day course of IV antibiotics collectively, followed by four days of oral clindamycin to complete a total duration of 14 days of antibiotics treatment on discharge.

Post-operatively, she showed significant clinical improvement with resolution of the fever and stridor and quick return to her normal diet. Inflammatory markers and WBC count trended normal by post-operative day 7. She had a complete recovery from all her signs and symptoms with no evidence of recurrence or additional infections in the subsequent six months.

## Discussion

DNIs are relatively uncommon in children. Adil et al. noted a rate of 4.6 per 100,000 children using the 2009 KIDS’ Inpatient Database in the United States of America [12]. Parapharyngeal abscesses tend to occur due to non-traumatic reasons in young children, such as a prior throat infection or an infection of odontogenic origin that has seeded into the deeper tissue structures and lymph nodes [13]. Clinical presentation and physical findings might be subtle and difficult to detect in young children. Fever may be the only sign at initial presentation [14]. The next most common finding reported is neck pain or mass with reduced neck movement, which can be missed if not noted by the parents or identified during the initial physical exam. As such, detection and diagnosis has the potential to be significantly delayed. Additional findings that increase the suspicion for DNIs include trismus, uvular deviation, and odynophagia, presenting with reduced oral intake and/or drooling. These cases require high clinical suspension to achieve early detection and avoid serious complications such as the airway compression noted in our case [14]. Additional potential complications include sepsis, mediastinitis, jugular vein thrombosis, cranial nerve dysfunction, cervical osteomyelitis, meningitis, and death [1].

In our case, the culture was obtained via needle aspiration through the oral cavity which raises the possibility of contamination. However,* G. adiacens* was the only bacteria isolated. In addition, it was initially cultivated via Vitek 2 Gram-Positive Identification Card (BioMérieux) and then confirmed by MALDI-TOF MS.

As reported in an extensive study ofG. adiancens susceptibility, only 38.9% of *G. adiacens* isolates were found susceptible to penicillin. However, a significant portion (47.8%) of isolates had intermediate susceptibility to penicillin. Sensitivity to other antibiotics included cefotaxime (18.9%), ceftriaxone (43.3%), erythromycin (52.2%), clindamycin (84.5%), and levofloxacin (91.9%), but all*G. adiacens* isolates were 100% sensitive to meropenem and vancomycin [3]. This profile was consistent with our case isolate’s susceptibility, which was resistant to penicillin and sensitive to vancomycin and clindamycin; both these latter antibiotics were used successfully to treat our patient after surgical drainage.

Regarding medical management of DNIs, antibiotics with or without surgicalintervention remain a mainstay in their treatment. In patients who show a positive clinical response to IV antibiotics, especially those with smaller abscesses size, high dose intravenous antibiotics without surgical drainage can be an effective treatment [1,15]. Literature has shown that children younger than 51 months of age, patients requiring intensive care unit admission and cases with CT findings consistent with abscess size greater than 2.2 cm in diameter are risk factors for failure of medical treatment [16].

On the other hand, Tansey et al. reported that children with DNIs who received dexamethasone (1 mg/kg/dose, up to 10 mg, every eight hours for 48 hours) are more likely to resolve on medical treatment without surgical intervention [17]. Moreover, steroids reduce airway edema in children with impending airway obstruction as well as improve trismus and torticollis [16,18,19]. Still, its effect on length of stay remains controversial [20]. The reported side effects of steroids were noted to be minimal in a systematic review of their role in cervicofacial infections [20]. However, a few case studies report administration of steroids alone without the appropriate antibiotic coverage can lead to severe complications [20]. The senior author (SK), does express some caution with this above protocol as the dexamethasone dose is quite high. Further research is needed on dexamethasone in pediatric parapharyngeal abscesses.

Imaging is an important tool to assist in diagnosis and surgical decision-making. Classically, due to their ease and accessibility, lateral neck X-rays were used to assess for increased pre-vertebral soft tissue thickening to diagnose retropharyngeal abscess. However, its margin of error is affected by incorrect positioning, respiratory phase, and anatomical variance of cervical lordosis. Neck flexion and expiratory views can lead to false apparent thickening of the retropharyngeal tissue. In fact, a systematic review by Daniel et al. found the sensitivity of lateral neck X-rays to range from 0 to 100%. On the other hand, the same review noted higher sensitivity and specificity in cases where the patients were severely unwell [21].

Contrast-enhance CT scanning is generally accepted as the imaging modality of choice to diagnose and characterize DNIs. However, the CT signs have variable accuracy in characterizing evolving abscess and cellulitis or phlegmon. While complete rim enhancement and scalloping of the wall of the lesion favors an abscess, signs such as low density central core, fat stranding, and mass effect can be observed both in abscess and phlegmon [22]. In clinical practice, this variability possesses a diagnostic dilemma. An ultrasound examination may add value as the demonstration of liquefaction and fluid motion within necrotic tissues supports the presence of an abscess [23]. CT provides additional information related to the size of the abscess cavity, extension of the abscess into adjacent neck spaces and structures. Recent literature by Eisa and Mehanna show that by using a dual phase-contrast CT examination, phlegmon and abscess can be more confidently differentiated; the dual-phase of intravenous contrast administration allows enhancement of the central portion of a phlegmon by a slow interstitial uptake, while an abscess demonstrates a rim enhancement sign of an abscess [24].

Pairing CT scan findings with the clinical picture and medical treatment already received are essential for determining surgical candidacy. Surgery should be considered in patients who do not respond to antibiotics within 48 hours of initiation. Virbalas and Friedman recommend surgical intervention after CT-confirmed abscess in patients with hemodynamic instability, immunosuppression, toxic appearance, signs of airway obstruction, chest pain or otherwise not able to tolerate a trial of medical management. For patients without the above symptoms, but with the presence of a lateral neck swelling, screening neck ultrasound modify** **can be considered [25]. In our case, the child had airway symptoms, but the family were hesitant to proceed directly to CT because of the risks associated with radiation and general anesthesia. Ultimately after the ultrasound, CT with contrast was completed, which then led to definitive surgical management.

## Conclusions

Anaerobic cultures to grow additional bacteria such as G. adiacens should be collected in pediatric patients with DNIs. Contrast-enhance CT is the imaging modality of choice to diagnose and characterise DNIs. Dexamethasone, when used, can aid improve the inflammatory process, reduce airway edema, and help increase the success rate of medical management.
